# The Passive Series Stiffness That Optimizes Torque Tracking for a Lower-Limb Exoskeleton in Human Walking

**DOI:** 10.3389/fnbot.2017.00068

**Published:** 2017-12-20

**Authors:** Juanjuan Zhang, Steven H. Collins

**Affiliations:** ^1^Department of Mechanical Engineering, Carnegie Mellon University, Pittsburgh, PA, United States; ^2^School of Electric and Electronic Engineering, Nanyang Technological University, Singapore, Singapore; ^3^College of Computer and Control Engineering, Nankai University, Tianjin, China; ^4^Robotics Institute, Carnegie Mellon University, Pittsburgh, PA, United States; ^5^Department of Mechanical Engineering, Stanford University, Stanford, CA, United States

**Keywords:** series elastic actuators, lower-limb exoskeletons, ankle foot orthosis, torque control, optimal passive stiffness

## Abstract

This study uses theory and experiments to investigate the relationship between the passive stiffness of series elastic actuators and torque tracking performance in lower-limb exoskeletons during human walking. Through theoretical analysis with our simplified system model, we found that the optimal passive stiffness matches the slope of the desired torque-angle relationship. We also conjectured that a bandwidth limit resulted in a maximum rate of change in torque error that can be commanded through control input, which is fixed across desired and passive stiffness conditions. This led to hypotheses about the interactions among optimal control gains, passive stiffness and desired quasi-stiffness. Walking experiments were conducted with multiple angle-based desired torque curves. The observed lowest torque tracking errors identified for each combination of desired and passive stiffnesses were shown to be linearly proportional to the magnitude of the difference between the two stiffnesses. The proportional gains corresponding to the lowest observed errors were seen inversely proportional to passive stiffness values and to desired stiffness. These findings supported our hypotheses, and provide guidance to application-specific hardware customization as well as controller design for torque-controlled robotic legged locomotion.

## 1. Introduction

Direct control of interaction forces or torques have been used in physical human-robot interaction to reduce interface impedance, increase reactiveness of the robotic devices and thus improves human safety and comfort (Haddadin et al., 2008; Lasota et al., 2014). Torque control also provides a simple means of manipulating the flow of energy from the exoskeleton to the human, which can be useful in biomechanics studies (Veneman et al., 2007; Sawicki and Ferris, 2009; Stienen et al., 2010; Malcolm et al., 2013; Jackson and Collins, 2015). It can also be used to exploit passive dynamics or render virtual systems with alternate dynamics in humanoid robots (Pratt et al., 1997), active prostheses (Au et al., 2008; Sup et al., 2009; Caputo and Collins, 2013), and exoskeletons (Kawamoto et al., 2010; Unluhisarcikli et al., 2011; Giovacchini et al., 2014; Witte et al., 2015). In exoskeletons and prostheses, torque control quality is a limiting factor in accuracy of the applied intervention or assistance and can be the limiting factor in system performance. Therefore, there are always motivations to improve torque control performance in these systems.

Various efforts have been made toward accurate torque tracking of lower-limb wearable robotic devices bym means of controller design (van Dijk et al., 2013; Zanotto et al., 2013; Zhang et al., 2015, 2017). This type of systems have complicated, time-varying and uncertain dynamics due to human-robot interactions and possible transmission frictions. Inter-subject and inter-step variations of human gait also introduce hard-to-quantify noise and disturbances. Such characteristics result in the effectiveness of a torque tracking structure made of the combination of model-free, integration-free feedback control and iterative learning (van Dijk et al., 2013; Zhang et al., 2015, 2017). With this control architecture, fairly strong interactions were also observed experimentally in torque tracking between the device passive stiffness and high-level controller that determines desired torques, which suggest potential room for further improvement of torque tracking performance. This paper explores the possibility to improve torque control performance in lower-limb exoskeletons under fixed control objectives, with limited knowledge of the complete system dynamics, by optimizing the passive stiffness of actuators.

Traditionally, robot actuation has been made as stiff as possible for improved precision, stability and bandwidth of position control (Salisbury et al., 1991; Pratt and Williamson, 1995). Compliant actuators have been widely used in wearable devices during the past two decades due to their ability to improve human safety and comfort (Zinn et al., 2004; de Luca et al., 2006; Haddadin et al., 2008; Ham et al., 2009; Lasota et al., 2014) and reduce shock loads (Pratt and Williamson, 1995; Pratt et al., 1997; Ham et al., 2009). In the realization of compliant actuation, passive compliance is usually involved due to its improved safety, increased interaction control bandwidth and its ability of energy storage (Whitney, 1987; Pratt et al., 1997; Ham et al., 2009). Series elastic actuators are one type of actuators with passive compliance that have gained great popularity due to their enhanced force control performance and improved human experience (Pratt and Williamson, 1995; Robinson et al., 1999; Pratt et al., 2004; Zinn et al., 2004; Sensinger et al., 2006; Wyeth, 2006).

The stiffness of the elastic element is crucial in design of series elastic actuators since it highly affects the performance of the device in various applications (Hollander et al., 2006). Multiple criteria have been employed in the selection of passive compliance. Higher spring stiffness increases the open-loop bandwidth of an actuator and is more desirable for the purpose of increasing force bandwidth (Paine et al., 2014). Lower stiffness, however, increases the capacity of energy storage in actuators (Paine et al., 2014). Many devices also select series stiffness by balancing torque/force requirements, device geometry and device weight (Kong et al., 2009; Sawicki and Ferris, 2009; Witte et al., 2015). Multiple works discussed the optimization of passive stiffness in series elastic actuators for fixed applications from the perspective of energy consumption (Hollander et al., 2006; Vanderborght et al., 2008, 2009). Some aimed to optimize total energy consumption by general analysis (Vanderborght et al., 2006, 2008, 2009), matching the natural frequency of the actuator to the desired motion (Jafari et al., 2013), or matching system source and load impedance (Ozawa et al., 2015). Some tried to optimize application peak power capacity (Hollander et al., 2006).

There were some previous works that addressed the effects of passive stiffness of series elastic actuators on torque tracking performance, most of which were by means of theory (Ham et al., 2009; Vanderborght et al., 2009), simulation or benchtop tests without realistic experimental conditions (Ozawa et al., 2015). Veneman et al. (2006) briefly discussed the role of series elastic actuator passive stiffness in torque tracking, which states that too high and too low stiffness both worsen its control performance. It would be beneficial to investigate thoroughly the existence of the optimum in passive stiffness and its exact value by means of both theoretical analysis and realistic experiments.

This paper attends to the optimization of the passive series stiffness in lower-limb exoskeletons in the context of torque tracking during human walking, under the popular impedance based high level controller (Sawicki and Ferris, 2009; Caputo and Collins, 2013; Witte et al., 2015) using both theory and experiments. This type of high level controller adjusts the torque applied to human body according to joint position and realizes a desired quasi-stiffness, which is defined as the slope of the desired torque-angle relationship (Rouse et al., 2013). It is especially popular among locomotion related robots due to the high repeatability and ease of energy input manipulation to human body (Sawicki and Ferris, 2009; Caputo and Collins, 2013; Witte et al., 2015). The results are expected to guide application-specific hardware design of lower limb wearable robots used in gait assistance or rehabilitation.

## 2. Methods

To investigate the influence of passive stiffness on torque tracking performance in series elastic actuators, we did a case study on a tethered ankle-foot exoskeleton driven by a uni-directional Bowden cable (Witte et al., 2015). We developed a simplified model of the exoskeleton system, base on which we made hypotheses that relate torque tracking performance, actuator passive stiffness and control parameters. We then conducted walking experiments with one subject wearing the exoskeleton on the right foot. Eight different desired torque-angle relationships were implemented. Each was tested in combination with six passive stiffness configurations by switching the series spring in the transmission sub-system of the device. Every combination of desired and passive stiffness values, which we denote as one “stiffness combination” hereinafter in this paper, incurred multiple walking experiments on treadmill while the subject wore the device with different control gains. Comparison of the resulting torque tracking errors of these experiments identifies the best-observed tracking performance and corresponding control parameters of the current stiffness combination, which serve as the estimates of the actual optimal performance and control parameters. The observed optima were then investigated against the values of desired and passive stiffnesses to test and validate the hypotheses.

### 2.1. Exoskeleton system and simplified model

**Figure 1:**
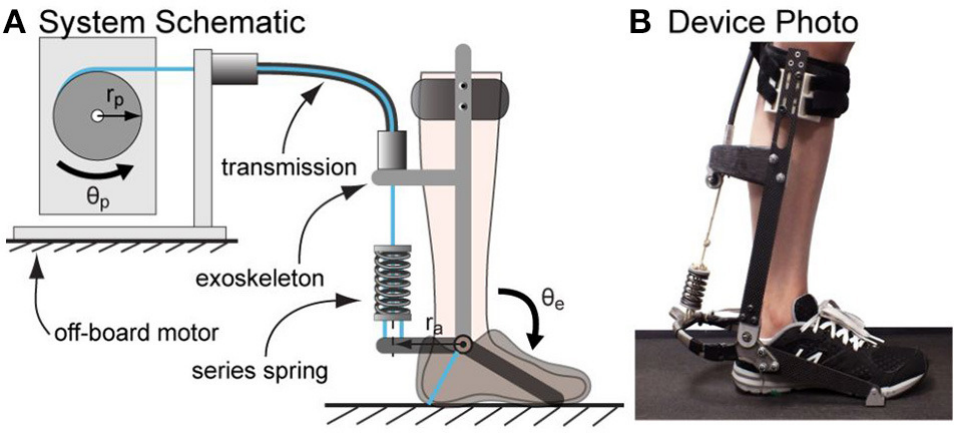
Tethered ankle exoskeleton system. **(A)** A schematic of the system that consists of an off-board motor system, a Bowden cable transmission ended with a series spring and an exoskeleton frame. For each desired quasi-stiffness, passive stiffness of the system was varied by switching the series spring and walking experiments were conducted to identify the optimal passive stiffness value. **(B)** A photo of the device worn by a subject standing on treadmill.

The exoskeleton testbed system comprised of an off-board real-time control module and geared electric motor, a uni-directional Bowden cable transmission with a series spring, and an ankle exoskeleton frame that interfaced with the human foot and shank (Figure 1).

To facilitate and ease mathematical analysis and make prediction of passive stiffness optimum for a pre-determined desired quasi-stiffness, the following simplifications were made in modeling the system:

1. There is zero friction in Bowden cable transmission, and the tension in the cable is the same at the motor output pulley and the ankle exoskeleton sides at any time. We define this tension as *F*.
2. The transmission sub-system consisting of the Bowden cable and the series spring behaves like a linear spring, i.e.,
  (1) $$\begin{array}{l} F=K_{c}\cdot \left(r_{p}\cdot \theta_{p}-r_{a}\cdot \theta_{e}\right) \end{array}$$
  in which *K*_*c*_ is the total effective stiffness of the Bowden cable transmission and series spring; θ_*p*_ and θ_*e*_ are the pulley and exoskeleton joint angles relative to a position at which the Bowden cable begins to go slack; *r*_*p*_ and *r*_*a*_ are the pulley radius and the lever arm at the ankle joint.
3. The range of motion of the exoskeleton joint is relatively small, which is around ±10 degrees around a nominal position. Therefore, the lever arm of the cable tension relative to the device joint, *r*_*a*_, is small and can be assumed constant.
  The torque at the exoskeleton side of the transmission can thus be expressed as
  (2) $$\begin{array}{l} \tau =F\cdot r_{a}. \end{array}$$

Combining Equations (1) and (2), we can express the torque applied by the exoskeleton to the human ankle joint as

(3) $$\begin{array}{l} \tau =F\cdot r_{a}\\ =r_{a}^{2}\cdot K_{c}\left[\theta_{p}\frac{r_{p}}{r_{a}}-\theta_{e}\right]\\ =K_{t}\left(\theta_{p}R-\theta_{e}\right). \end{array}$$

in which the aspect ratio of transmission is

(4) $$\begin{array}{l} R=\frac{r_{p}}{r_{a}}, \end{array}$$

and the transmission stiffness *K*_*t*_ which relates torque applied by the exoskeleton to the device joint angle is defined as

(5) $$\begin{array}{l} K_{t}=r_{a}^{2}\cdot K_{c}. \end{array}$$

### 2.2. Controllers

#### 2.2.1. Low level control: proportional control + damping injection

Previous work identified model-free, integral-action-free feedback control compensated by iterative learning as the most effective controller for lower-limb exoskeletons torque tracking during walking (Zhang et al., 2015, 2017). To simplify the testing of tracking performance, only the feedback part of the controller was used in theoretical analysis and experiments of this study:

(6) $$\begin{array}{l} {\dot{\theta }}_{p,des}=\underset{\text{Proportional Control}}{\underset{︸}{-K_{p}\cdot e_{\tau }}}+\underset{\text{Damping Injection}}{\underset{︸}{-K_{d}\cdot {\dot{\theta }}_{p}}}\\ {\dot{\theta }}_{m,des}=N{\dot{\theta }}_{p,des} \end{array}$$

This controller has two parts: proportional control and damping injection. In this formulation, *e*_τ_ = τ − τ_*des*_ is torque error, τ is measured exoskeleton torque, τ_*des*_ is desired exoskeleton torque. Damping injection on motor velocity is used instead of derivative control over torque errors to reduce the effect of measurement noise, which is more severe in the latter since torque is measured and transmitted in analog form while motor position is in digital and non-linearities is present in Bowden cable transmission. *K*_*p*_ is a proportional gain and *K*_*d*_ is a damping gain. The motor runs in velocity mode with the desire motor output pulley velocity ${\dot{\theta }}_{p,des}$ converted to desired motor velocity ${\dot{\theta }}_{m,des}$ before asserted. *N* is the gear ratio of the motor.

#### 2.2.2. High level control: desired quasi-stiffness

**Figure 2:**
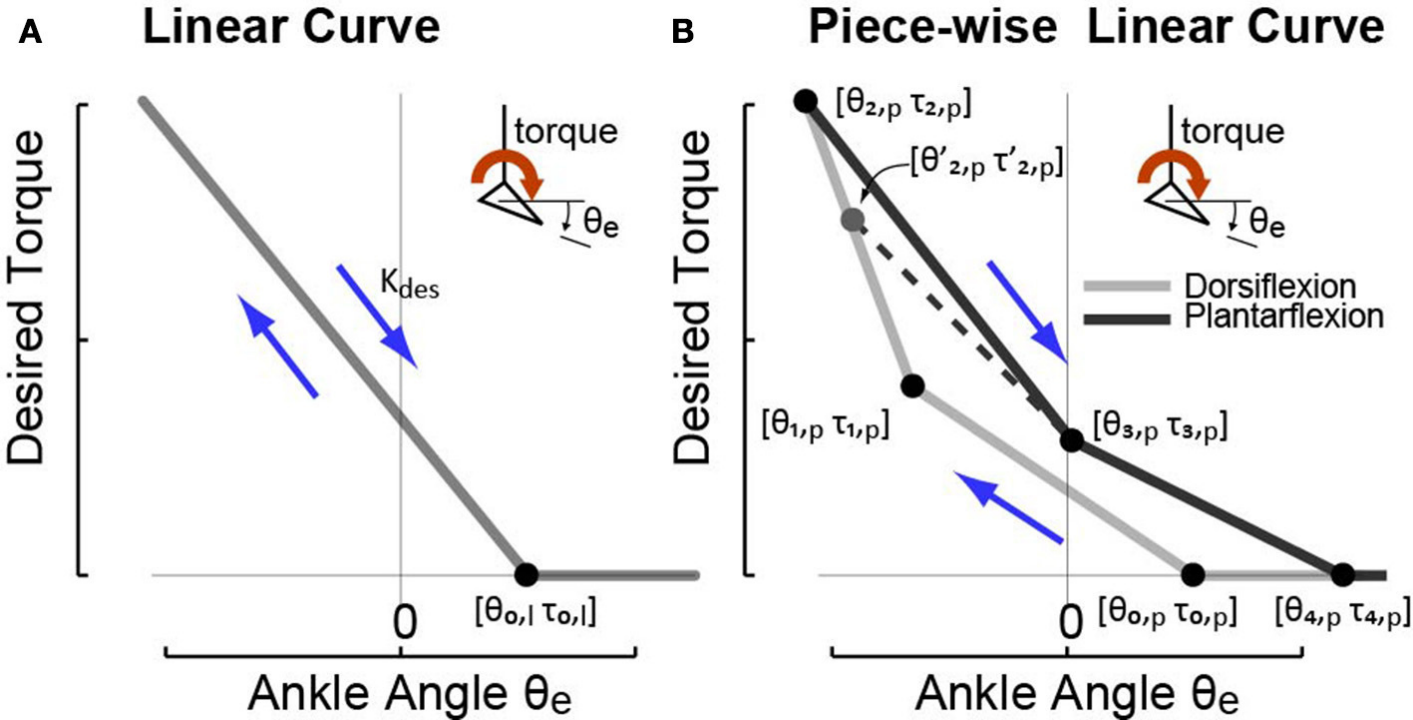
The two types of ankle angle based high-level desired torque curves imposed in experiments to realize different desired quasi-stiffness profiles. **(A)** High-level controller I commands desired torque that is linearly proportional to exoskeleton joint angle θ_*e*_ defined by anchor point [θ_0,*l*_ τ_0,*l*_] and desired quasi-stiffness *K*_*des*_. **(B)** High-level controller II commands desired torque that is piece-wise linearly proportional to joint angle θ_*e*_ with four phases defined by anchor points [θ_0,*p*_ τ_0,*p*_], [θ_1,*p*_ τ_1,*p*_], [θ_2,*p*_ τ_2,*p*_], [θ_3,*p*_ τ_3,*p*_], and [θ_4,*p*_ τ_4,*p*_]. In both cases, desired torque is lower-bounded at zero.

Two types of ankle angle based desired torque curves were used to realize different desired quasi-stiffnesses. One was a linear torque vs. ankle angle curve as shown in Figure 2A and expressed in Equation (7).

(7) $$\begin{array}{l} \tau_{des}=S\cdot K_{des,0}\cdot \left(\theta_{e}-\theta_{0,l}\right)\\ =-K_{des}\cdot \left(\theta_{e}-\theta_{0,l}\right)\\ \tau_{des}=\max\left(\tau_{des},0\right) \end{array}$$

where (θ_0,*l*_, τ_0,*l*_) is an anchor node in torque-angle space and *K*_*des*_ is the resulted desired stiffness. *S* is a scaling factor on the unit curve with desired quasi-stiffness of *K*_*des*, 0_ to get different values of desired quasi-stiffness.

Another type of curve tested was a piece-wise linear torque-angle curve with the format of Equation (8) as shown in Figure 2B.

(8) $$\begin{array}{l} \tau_{des}=S\cdot \frac{\tau_{i,p}-\tau_{i-1,p}}{\theta_{i,p}-\theta_{i-1,p}}\cdot \left(\theta_{e}-\theta_{i-1,p}\right)\\ \tau_{des}=\max\left(\tau_{des},0\right)\\ \text{for }i=\left\{1,2,3,4\right\}, \end{array}$$

Here, (θ_*i,p*_, τ_*i,p*_) defines a node in torque-angle space (Figure 2B). *i* denotes the phase number on the curve the gait is currently in. The node (θ_2,*p*_, τ_2,*p*_) marked the transition from the dorsiflexion phase, in which ankle velocity was negative, to the plantarflexion phase, in which ankle velocity was positive. Since the exact transition point varied on each stride, we used the angle and torque at the moment of transition, $\left(\theta_{2,p}^{\prime },\tau_{2,p}^{\prime }\right)$, when calculating desired torque in the first portion of Plantarflexion, i.e., phase 3 of the curve.

Note that the piece-wise linear curve mimics the positive work loop produced by human ankle torque-angle relationship during walking, which was commonly used by lower-limb exoekeletons and prostheses in high level control (Caputo and Collins, 2013). The linear model is a special case for the piec-wise linear model, and a simplified version, which will be used in theoretical analysis later in this article.

### 2.3. Theoretical analysis and hypotheses

We conducted theoretical analysis based on the analytic expressions of the testbed system dynamics, desired torque, and torque controller and made hypotheses about the optimum of passive stiffness of series elastic actuators in lower-limb ankle exoskeletons and the interactions between optimal gains, desired stiffness and passive stiffness.

#### 2.3.1. Optimal passive stiffness

To further ease the theoretical analysis for the prediction of passive stiffness optimum in series elastic actuators, we modeled the assisted walking with the ankle exoskeleton as an oscillator. Oscillators are efficient modeling tools in biological and physical sciences due to their capability to synchronize with other oscillators or with external driving signals (Collins and Stewart, 1993b; Righetti et al., 2009). Multiple efforts have been made toward improving the synchronization capabilities of non-linear oscillators by adapting their frequencies (Righetti et al., 2009). The concept has been introduced and employed in locomotion to either improve the identification of central pattern generator parameters (Bay and Hemami, 1987; Collins and Stewart, 1993a,b; Buchli et al., 2006; Morimoto et al., 2006), to better estimate state measurements (Dutra et al., 2003; Ronsse et al., 2011; van Dijk et al., 2013), or to help with controller design (Tsuchiya et al., 2003; Aoi and Tsuchiya, 2005) by exploiting the cyclic behavior of walking. Therefore, we model various states of walking as synchronized oscillations. This method disburden our analysis from dealing with complicated human-robot interactive dynamics, focus on the resulting states like ankle kinematic profile and required motor position profile that are close to be periodical, and significantly simplified our analysis. However, neglecting of step-to-step variations in practical cases does cause potential deviation of experimental results from theoretical hypotheses.

With proportional control and damping injection in Equation (6) used for torque tracking,

(9) $$\begin{array}{l} {\dot{\theta }}_{p,des}=-K_{p}e_{\tau }-K_{d}{\dot{\theta }}_{p}\\ =-K_{p}\left[K_{t}\left(\theta_{p}R-\theta_{e}\right)+K_{des}\left(\theta_{e}-\theta_{0}\right)\right]-K_{d}{\dot{\theta }}_{p} \end{array}$$

Due to the employment of a high-speed real-time controller and a high-acceleration servo motor, desired motor velocity is enforced rapidly, based on which we make the simplification of immediate motor velocity enforcement, i.e.,

(10) $$\begin{array}{l} {\dot{\theta }}_{p}={\dot{\theta }}_{p,des}. \end{array}$$

Combining Equation (10) with a linear approximation of desired torque curves, including those expressed by Equations (7) and (8), in the form of

(11) $$\begin{array}{l} \tau_{des}=-K_{des}\left(\theta_{e}-\theta_{0}\right), \end{array}$$

we have

(12) $$\begin{array}{l} \left(1+K_{d}\right){\dot{\theta }}_{p}=-K_{p}\left[K_{t}\left(\theta_{p}R-\theta_{e}\right)+K_{des}\left(\theta_{e}-\theta_{0}\right)\right]. \end{array}$$

in which θ_0_ is maximum joint position for the device to exert torque on the human ankle, i.e., the intersection of torque-angle relationship with the angle axis.

Modeling exoskeleton-assisted walking after stabilization as an oscillation process made of *N* sinusoidal waves of the same frequency *F*, we get a profile of the ankle angle in the form of

(13) $$\begin{array}{l} \theta_{e}=c+\overset{N}{\underset{n=1}{\sum }}d_{n}\cdot \exp\left(j2\pi Ft+\beta_{n}\right), \end{array}$$

where *c* is an constant denoting the offset of the profile on torque axis, *d*_*n*_ and β_*n*_ are the magnitude and phase shift of the *n*th sinusoidal wave, and *t* represents the time elapsed within one stride since heel strike. The corresponding stabilized motor position should also oscillates with the same frequency. We can therefore construct a stabilized motor position by equal number of sinusoidal waves with the same phase shifts in the form of

(14) $$\begin{array}{l} \theta_{p}=e+\overset{N}{\underset{n=1}{\sum }}f_{n}\cdot \exp\left(j2\pi Ft+\beta_{n}\right), \end{array}$$

in which *e* is a constant and *f*_*n*_ is a complex number. Substituting Equations (13) and (14) into Equation (12), we get Equation (15).

(15) $$\begin{array}{l} \left[\left(1+K_{d}\right)j2\pi F+K_{p}K_{t}R\right]\overset{N}{\underset{n=1}{\sum }}f_{n}\cdot \exp\left(j2\pi Ft+\beta_{n}\right)\\ =-K_{p}K_{t}Re-K_{p}\left(K_{des}-K_{t}\right)c+K_{p}K_{des}\theta_{0}\\ -K_{p}\left(K_{des}-K_{t}\right)\overset{N}{\underset{n=1}{\sum }}d_{n}\cdot \exp\left(j2\pi Ft+\beta_{n}\right) \end{array}$$

Equating the coefficients of the various sinusoidal waves and the offset, we have

(16) $$\begin{array}{l} f_{n}=\frac{-K_{p}\left(K_{des}-K_{t}\right)}{\left(1+K_{d}\right)j2\pi F+K_{p}K_{t}R}d_{n} \end{array}$$

and

(17) $$\begin{array}{l} e=-\frac{K_{des}-K_{t}}{K_{t}R}c+\frac{K_{des}}{K_{t}R}\theta_{0}. \end{array}$$

Motor position profile in Equation (14) can thus be expressed in terms of the ankle position profile and the controller as

(18) $$\begin{array}{l} \theta_{p}=-\frac{K_{des}-K_{t}}{K_{t}R}c+\frac{K_{des}}{K_{t}R}\theta_{0}\\ +\frac{-K_{p}\left(K_{des}-K_{t}\right)}{\left(1+K_{d}\right)j2\pi F+K_{p}K_{t}R}\overset{N}{\underset{n=1}{\sum }}d_{n}\cdot \exp\left(j2\pi Ft+\beta_{n}\right). \end{array}$$

Combining the oscillator assumption with Equation (12), we get the expression of the torque error as

(19) $$\begin{array}{l} e_{\tau }\\ =\tau -\tau_{des}\\ =K_{t}\left(\theta_{p}R-\theta_{e}\right)+K_{des}\left(\theta_{e}-\theta_{0}\right)\\ =K_{t}R\theta_{p}+\left(K_{des}-K_{t}\right)\theta_{e}-K_{des}\theta_{0}\\ =\left(K_{des}-K_{t}\right)\frac{j2\pi F}{j2\pi F+\frac{K_{p}}{1+K_{d}}K_{t}R}\overset{N}{\underset{n=1}{\sum }}d_{n}\cdot \exp\left(j2\pi Ft+\beta_{n}\right) \end{array}$$

It is clear that without considering the control gains, asserting

$$K_{des}-K_{t}=0$$

will minimize torque tracking error. Therefore, we make the following hypothesis:

**Hypothesis 1**. *In lower-limb exoskeletons, the optimal passive stiffness of the series elastic actuator for torque tracking is*

(20) $$\begin{array}{l} K_{t,opt}=K_{des} \end{array}$$

#### 2.3.2. Relationship between torque tracking performance and the difference of desired and passive stiffnesses

Another factor that limits torque tracking performance is the inability of the proportional gain to increase indefinitely.

Reformating Equation (19), we get

(21) $$\begin{array}{l} e_{\tau }=\\ \frac{j2\pi F}{\frac{K_{p}}{1+K_{d}}K_{t}R+j2\pi F}\left(K_{des}-K_{t}\right)\overset{N}{\underset{n=1}{\sum }}d_{n}\cdot \exp\left(j2\pi Ft+\beta_{n}\right). \end{array}$$

It is clear that when the passive stiffness is fixed but does not match the desired one, i.e., *K*_*t*_ − *K*_*des*_ ≠ 0, with the same step frequency *F* and angle profile $\overset{N}{\underset{n=1}{\sum }}d_{n}\cdot \exp\left(j2\pi Ft+\beta_{n}\right)$, torque tracking error *e*_τ_ is inversely proportional to $\frac{K_{p}}{1+K_{d}}$.

Meanwhile, combining the controller in Equation (9) and the assumption of perfect motor velocity tracking in Equation (10), we have

(22) $$\begin{array}{l} {\dot{\theta }}_{p}=-\frac{K_{p}}{1+K_{d}}e_{\tau } \end{array}$$

Differentiating the expression of applied torque in Equation (3), we get

(23) $$\begin{array}{l} \dot{\tau }=K_{t}\left({\dot{\theta }}_{p}R-{\dot{\theta }}_{e}\right) \end{array}$$

Therefore, the time derivative of torque error is

(24) $$\begin{array}{l} {ė}_{\tau }=\dot{\tau }-{\dot{\tau }}_{des}\\ =K_{t}\left({\dot{\theta }}_{p}R-{\dot{\theta }}_{e}\right)+K_{des}{\dot{\theta }}_{e}\\ =-K_{t}R\frac{K_{p}}{1+K_{d}}e_{\tau }-K_{t}{\dot{\theta }}_{e}+K_{des}{\dot{\theta }}_{e} \end{array}$$

which is a first order dynamics created by feedback control with an effective proportional gain of

$$\frac{K_{t}\cdot R\cdot K_{p}}{1+K_{d}}$$

and a time constant of

$$\varsigma =\frac{1+K_{d}}{K_{t}\cdot R\cdot K_{p}}.$$

However, this dynamics does not exist independently but interacts with the human body in parallel. Therefore, in practical cases, oscillations increase when effective proportional gain increases, which impairs torque tracking performances eventually and causes discomfort or injury to the human body. In our study, motor speed limit was never hit. Thus, we suspected that there is a fixed torque tracking bandwidth limit that is dependent on the combined interactive dynamics of motor, motor drive, transmission and human body. This bandwidth limit results in a fixed maximum commanded change rate of torque error, ė_τ, max_, which corresponding to a best tracking performance regardless of the passive stiffness of the system. We therefore proposed the following conjecture:

**Conjecture 1**. *Assisted human walking with a lower-limb exoskeleton experiences a fixed maximum commanded tracking rate of torque error, ė*_τ,*max*_, *which limits the tracking performance of the system*.

In practical cases, Equation (24) can be further simplified. First, to realize real-time torque tracking, the motor velocity should be a lot faster then device joint velocity, i.e., ${\dot{\theta }}_{p}\gg {\dot{\theta }}_{e},$ which combines with the fact that *R* = 2.5 results in the following fact about Equation (23):

(25) $$\begin{array}{l} \dot{\tau }\approx K_{t}R{\dot{\theta }}_{p}. \end{array}$$

Successful torque tracking also means a fast changing rate of actual torque compared to the desired torque, $\dot{\tau }\gg {\dot{\tau }}_{des}$, which leads to the results of dominance of applied torque changing rate in torque error changing rate, i.e.,

(26) $$\begin{array}{l} {ė}_{\tau }\approx \dot{\tau } \end{array}$$

Therefore, Equation (24) can be estimated as:

(27) $$\begin{array}{l} {ė}_{\tau }\approx -K_{t}R\frac{K_{p}}{1+K_{d}}e_{\tau } \end{array}$$

According to Conjecture, there is a fixed maximum ė_τ_ which corresponding to optimal control performance. In this case, with comparable torque error *e*_τ_, $\frac{K_{p}}{1+K_{d}}$ and *K*_*t*_ are inversely proportional to each other. In other words, the application of Conjecture 1 results in a fixed time constant $\frac{1+K_{d}}{K_{t}RK_{p}}$ at optimal control conditions. Together with the assumption of a rather constant step frequency *F* and a constant angle profile $\overset{N}{\underset{n=1}{\sum }}d_{n}\cdot \exp\left(j2\pi Ft+\beta_{n}\right)$, torque error as expressed by Equation (21) is proportional to the difference between passive and desired stiffness values, i.e.,

$$e_{\tau ,opt}\propto K_{des}-K_{t}.$$

Then the root-mean-squared value of *n* instanteneous torque tracking errors at optimal conditions can be expressed as

$$\begin{array}{c} e_{\tau ,opt,RMS}=\sqrt{\frac{e_{\tau ,opt}{\left(t_{1}\right)}^{2}+e_{\tau ,opt}{\left(t_{2}\right)}^{2}+\ldots +e_{\tau ,opt}{\left(t_{n}\right)}^{2}}{n}}\\ =\sqrt{\frac{C_{1}{\left(K_{des}-K_{t}\right)}^{2}+C_{2}{\left(K_{des}-K_{t}\right)}^{2}+\ldots +C_{n}{\left(K_{des}-K_{t}\right)}^{2}}{n}}\\ =\sqrt{\frac{C_{1}+C_{2}+\ldots +C_{n}}{n}}||K_{des}-K_{t}|| \end{array}$$

in which *C*_1_, *C*_2_, …, *C*_*n*_ are some constant coefficients. This then leads to the hypothesis below.

**Hypothesis 2**. *The root-mean-squared torque tracking errors under optimal feedback control conditions are proportional to the absolute difference between the desired and passive stiffness values, i.e.*,

(28) $$\begin{array}{l} ||e_{\tau ,opt,RMS}||\propto ||K_{des}-K_{t}||. \end{array}$$

#### 2.3.3. Interactions between optimal control gains and passive stiffness

Dynamics in Equation (24) directly leads to a relationship between *K*_*p*_ and *K*_*t*_:

(29) $$\begin{array}{l} K_{p}=\frac{\left(K_{des}{\dot{\theta }}_{e}-{ė}_{\tau }\right)\left(1+K_{d}\right)R^{-1}e_{\tau }^{-1}}{K_{t}}-{\dot{\theta }}_{e}\left(1+K_{d}\right)R^{-1}e_{\tau }^{-1}. \end{array}$$

which can be simplified under the same desired torque-angle relationship, i.e., *K*_*des*_. Previous study shows an root-mean-squared tracking error of <8% the peak desired torque under proportional control and damping injection (Zhang et al., 2015, 2017), which is expected to be improvable with better control parameters and different curve types. This suggests that under optimal torque tracking conditions, the actual applied torque profiles with the same *K*_*des*_, are expected to be fairly constant regardless of the value of passive stiffness *K*_*t*_. Meanwhile, although the exact exoskeleton-human interactive dynamics is difficult to identify, we expect the relationship between applies torque and resulting human ankle kinematics to obey of Newton's law. Therefore, a fairly constant torque profile from the exoskeleton, when applied to the same subject under the same walking speed and step frequencies with low variance, should produce rather constant human and device joint kinematics, θ_*e*_ and ${\dot{\theta }}_{e}$. Therefore, the extreme device joint velocity that would produce the highest torque error rate with fixed control gains and push the controlled system to its bandwidth limit, θ_*e,ext*_, does not vary significantly across different passive stiffness conditions. Similar assumptions can be made about the extreme torque error *e*_τ,*ext*_. On the other hand, gain of the less dominant damping injection control part, *K*_*d*_, have been observed to be upper-bounded by the appearance of motor juddering in our experiments at *K*_*d,max*_ = 0.6 for various stiffness combinations. The approximated invariance of θ_*e,ext*_ and *K*_*d,max*_, combined with a fixed ė_τ,max_ as assumed by Conjecture 1, lead to the following hypothesis.

**Hypothesis 3**. *With the same desired torque-angle curve, thus the same K_des_, the optimal proportional gain K_p,opt_ is related to the passive stiffness K_t_ by*

(30) $$\begin{array}{l} K_{p,opt}=\frac{\sigma }{K_{t}}+\lambda , \end{array}$$

*in which* σ *is dependent on the desired stiffness K_des_ and can be expressed as*

(31) $$\begin{array}{l} \sigma =\left(K_{des}{\dot{\theta }}_{e,ext}-{ė}_{\tau ,max}\right)\left(1+K_{d,max}\right)R^{-1}e_{\tau ,ext}^{-1} \end{array}$$

*and the constant* λ *is*

(32) $$\begin{array}{l} \lambda =-{\dot{\theta }}_{e,ext}\left(1+K_{d,max}\right)R^{-1}e_{\tau ,ext}^{-1} \end{array}$$

To ease later presentation, we label the value σ here as K_p_ − K_t_ **coefficient** hereinafter.

On the other hand, to realize torque tracking, proportional control is always dominant over damping injection. Therefore, Equation (22) can be simplified as

(33) $$\begin{array}{l} {\dot{\theta }}_{p,des}\approx -K_{p}e_{\tau } \end{array}$$

and accordingly, Equation (27) becomes

(34) $$\begin{array}{l} {ė}_{\tau }\approx -K_{t}RK_{p}e_{\tau } \end{array}$$

which suggests that we can further simplify Hypothesis 3 with an approximated inverse proportional relationship between the optimal *K*_*p*_ and *K*_*t*_. According to Conjecture 1, at optimal conditions, ė_τ_ is fixed. With comparable torque error *e*_τ_, the following corollary can be made.

**Corollary 1**. *For a fixed desired torque-angle relationship, i.e, K_des_, when the passive stiffness of the series elastic actuator of the device is changed from K_t,old_ to K_t,new_, an estimate of the new optimal proportional control, K_p,new_, can be achieved by*

(35) $$\begin{array}{l} K_{p,\text{new}}\approx \frac{K_{p,\text{old}}\cdot K_{t,\text{old}}}{K_{t,\text{new}}}. \end{array}$$

*in which K_p,old_ is the optimal at K_t,old_*.

Although multiple approximations have been made in the derivation of this corollary, which causes inaccuracies in this estimation, it can be used to set a starting point of proportional control gain tuning when system passive stiffness is changed with only the knowledge of the old and new passive stiffness values.

#### 2.3.4. Relationship between *K*_*p*_ − *K*_*t*_ coefficient and desired stiffness

Furthermore, combining Equations (24), (30), (32) at optimal control conditions, we have

(36) $$\begin{array}{l} {ė}_{\tau ,max}=-\sigma R{\left(1+K_{d,max}\right)}^{-1}e_{\tau }+K_{des}{\dot{\theta }}_{e}\\ =-\sigma R{\left(1+K_{d,max}\right)}^{-1}\left[\tau +K_{des}\left(\theta_{e}-\theta_{0}\right)\right]+K_{des}{\dot{\theta }}_{e} \end{array}$$

which means

(37) $$\begin{array}{l} \sigma =\frac{\left(1+K_{d,max}\right)\left(K_{des}{\dot{\theta }}_{e}-{ė}_{\tau ,max}\right)}{R\left[K_{des}\left(\theta_{e,ext}-\theta_{0}\right)+\tau \right]} \end{array}$$

With relatively invariant extreme ankle velocity values, θ_*e,ext*_(*t*), and torque error values ė_τ,*max*_, across different desired stiffness, at a time of similar measured torque τ, the following hypothesis can then be drawn.

**Hypothesis 4**. *The K_d_* − *K_t_ coefficient in Equation (30) is related to the desired quasi-stiffness K_des_ by*

(38) $$\begin{array}{l} \sigma =\frac{\varsigma \cdot K_{des}+\delta }{K_{des}+\xi }, \end{array}$$

*in which* ς, δ *and* ξ *are constant parameters, and*

(39) $$\begin{array}{l} \delta =\frac{\left(1+K_{d,max}\right)}{R\left(\theta_{e,ext}-\theta_{0}\right)}{ė}_{\tau ,\text{max}}, \end{array}$$

*is linearly related to the hypothesized maximum commanded torque change rate* ė_τ, *max*_.

Combining Hypotheses 3 and 4, we can relate the optimal proportional gain to the desired quasi-stiffness and get the following corollary.

**Corollary 2**. *The optimal proportional control gain K_p,opt_ has an inverse linear relationship with the desired quasi-stiffness K_des_*.

Hypothesis 3 and Corollary 2 together suggest the monotonical decreasing trends of the optimal proportional control gain *K*_*p,opt*_ as either the passive stiffness *K*_*t*_ or the desired quasi-stiffness *K*_*des*_ increases.

### 2.4. Dynamic complications not featured

Multiple assumptions and approximations of the system dynamics were made to simplify our theoretical analysis in forming the hypotheses. Complications of the system that were not captured by the models we used are listed below.

1. There are frictions and stictions in the Bowden cable transmission, which are also time-varying depending on the shape of the cable, conditions of the inner rope and conduit, tension in the rope and also velocity of the relative motion between the rope and conduit.
2. The transmission stiffness is non-linear due to the existence of synthetic rope in Bowden cable, which is stretchy under a small tension and stiffer under larger. This stiffness is also subject to changes due to the slow-stretching of the rope and step-to-step gait variation.
3. The lever arm of series spring force with respect to the device joint is not a constant.
4. There is a communication delay and rise time during enforcement of the desired velocity. Therefore, there can never be perfect tracking.
5. Due to step-to-step variation, even after stabilization, the assisted walking is not an exactly periodical oscillatory process as assumed.

All these unfeatured complications in system dynamics can cause reality to deviate from the theoretical analysis to some extent. To ensure the guidance for hardware and controller design we provide to be meaningful practically, we conducted walking experiments to test and validate our hypotheses.

### 2.5. Testbed system configurations

We tested the hypotheses with different springs on a tethered ankle exoskeleton comprised of an off-board real-time control module and geared electric motor, a uni-directional Bowden cable transmission with a series spring, and an exoskeleton frame that interfaced with the human foot and shank (Figure 1).

In the tethered ankle exoskeleton testbed as shown in Figure 1, a dedicated real-time control system (ACE1103, dSPACE Inc.) sample sensors at 5,000 Hz, filter sensor data at 200 Hz, and generate control commands at 500 Hz. The motor unit was composed of a low-inertia 1.6 kW AC servo motor and a 5:1 planetary gear, with input voltage regulated by a motor driver running in velocity control mode (BSM90N-175AD, GBSM90-MRP120-5 and MFE460A010B, Baldor Electric Co.). A digital optical encoder (E5, US Digital Corp.) measured motor position.

The exoskeleton frame applied forces on the front of the human shank below the knee, beneath the heel, and beneath the toe, so as to generate an ankle plantarflexion torque in proportion to transmission force. Torque was measured using strain gauges (MMF003129, Micro-Measurements) applied in a full Wheatstone bridge on the heel lever, with 1,000 Hz signal conditioning (CSG110, Futek Inc.). Joint angle was measured using a digital optical encoder (E5, US Digital Corp.).

A flexible uni-directional Bowden cable transmitted forces from the motor to the exoskeleton frame while minimally restricting leg motions. The cable was composed of a coiled-steel outer conduit (415310-00, Lexco Cable Mfg.) and a 0.003 m diameter Vectran^Ⓡ^ inner rope, and was 2 m in length. A series spring was attached at the end of the rope to provide increased compliance. This spring is switched in tests to investigate the effects of changing passive stiffness on torque tracking in the device.

### 2.6. Experimental methods

The purpose of experiments in this study was to quantify not the human reaction but the torque tracking performance of various hardware and control conditions, in detail, the relative performance of torque tracking under different desired and passive stiffness conditions for the same subjects. In theoretical analysis, we conjected that the subject does not affect the relative relationship between desired/passive stiffness and tracking performance, but only the absolute values of the optimal control gains, and optimal tracking errors with a maximum tolerable exoskeleton control bandwidth (torque tracking rate). If this conjecture, together with the resulting hypotheses, is validated by single-subject experiments, then there is no need to test multiple subjects for the proof of the relative peroformance of different desired and passive stiffness values, which is unaffected by the subjects. If the conjecture and hypotheses do not even agree with single-sugject experiments, there is no meaning testing more subjects. Therefore, only one healthy subject (*N* = 1, female, 32 years, 1.65 m, 56 kg) was involved. The subject walked on a treadmill with a fixed speed of 1.25 m/s with a self-paced step frequency while wearing the tethered ankle exoskeleton on the right leg in all experiments. All experimental protocols were approved by Carnegie Mellon University IRB.

For the ease of readability, this paper uses Newton-meter, degree and meter as the corresponding units for measurements of torque, angle and distance.

#### 2.6.1. Desired torque-angle curves and evaluation of their desired stiffness values

**Figure 3:**
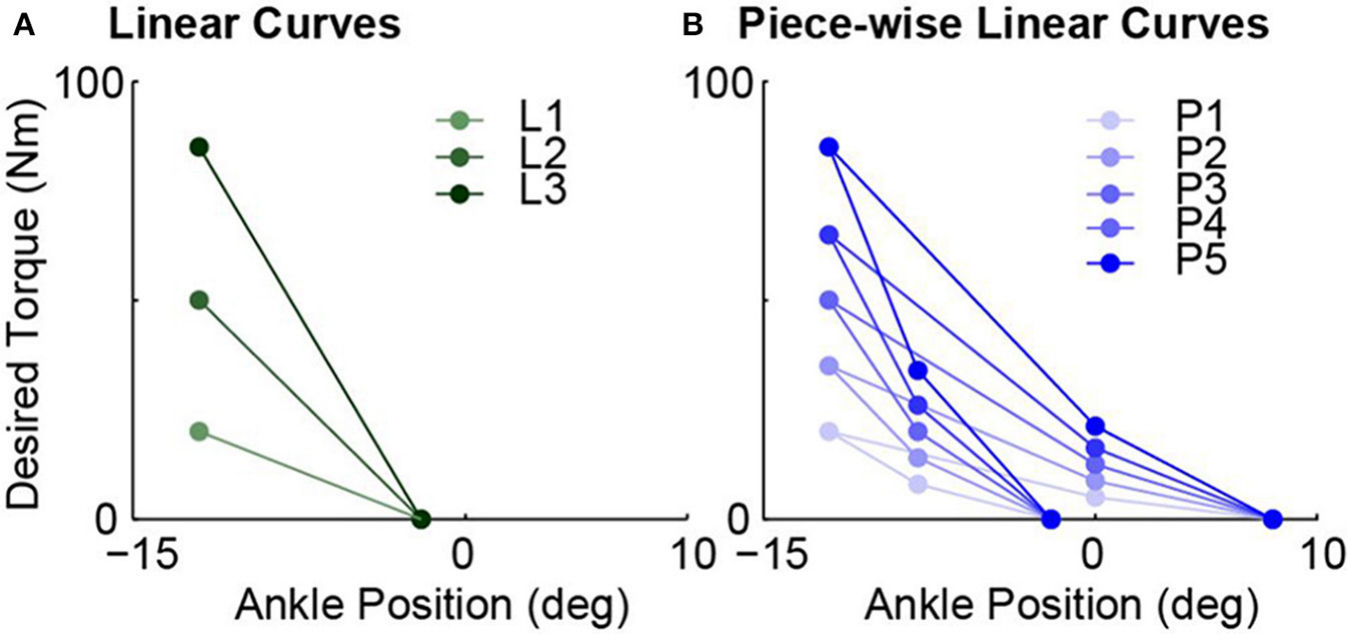
All eight tested desired torque vs. ankle angle curves used to realize different desired quasi-stiffness values. **(A)** Three linear curves achieved by scaling unit curve defined by Table 1 with scaling factors of 0.4, 1, and 1.7. **(B)** Five piece-wise linear curves by achieved by scaling unit curve defined by Table 2 with scaling factors of 0.4, 0.7, 1, 1.3, and 1.7.

To test the hypotheses, eight desired quasi-stiffnesses, i.e., torque vs. ankle angle relationship, were implemented, including three linear and five piece-wise linear curves. A unit linear curve (*S* = 1 in Equation 7) was defined by parameter values in Table 1. The three linear curves, L1, L2 and L3, were achieved by scaling the unit curve on the desired torque axis with factors of 0.4, 1, and 1.7 respectively. On the other hand, a unit piece-wise linear curve (*S* = 1 in Equation 8) was defined by the parameter values listed in Table 2. Five piece-wise linear curves, P1, P2, P3, P4 and P5, were then achieved by scaling the unit curve with factors 0.4, 0.7, 1, 1.3, and 1.7. The resulting desired torque vs. ankle angle curves are shown in Figure 3.

**Table 1:** Linear unit curve parameter values.

**Param**	**Value**	**Param**	**Value**
[θ_0,*l*_ τ_0,*l*_]	[−2, 0]	*K*_*des*,0_	5

**Table 2:** Piece-wise linear unit curve parameter values.

**Param**	**Value**	**Param**	**Value**
[θ_0,*p*_ τ_0,*p*_]	[−2, 0]	[θ_1,*p*_, τ_1,*p*_]	[−8, 20]
[θ_2,*p*_, τ_2,*p*_]	[−12, 50]	[θ_3,*p*_, τ_3,*p*_]	[0, 12.5]
[θ_4,*p*_, τ_4,*p*_]	[8, 0]		

Calculation of desired quasi-stiffness values are different for linear and piece-wise cases. For linear curves, the values of L1, L2, and L3 can be easily evaluated as 2, 5, and 8.5 Nm/deg respectively. This set spans a range of 6.5 Nm/deg with a maximum that is 4.25 times the minimum. For the case of piece-wise linear curves, we primarily used the desired stiffness values of each of the four phases and investigated different phases separately. The desired quasi-stiffness values in this case ranges from 0.625 to 12.75 Nm/deg.

#### 2.6.2. Realization of different passive stiffness and evaluation of their values

For each of the desired stiffness profile defined by a torque-angle relationship, six passive series stiffness values of the transmission system were realized by changing the series spring of the ankle exoskeleton (Figure 1A). Five of them were achieved by attaching different compression springs (Diamond Wire Spring, Glenshaw, PA) at the end of the series elastic actuators. One was realized by getting rid of the spring in the structure, in which case the system passive stiffness is solely determined by the stiffness of the synthetic rope in Bowden cable. The list of springs used and their corresponding properties are available in Table 3.

**Table 3:** List of springs used in experiments with assigned ID.

**Passive stiffness ID**	**S1**	**S2**	**S3**	**S4**	**S5**	**S6**
Spring Part No.	DWC-148M-13	DWC-162M-12	DWC-187M-12	DWC-225M-13	DWC-250M-12	No Spring
Length (m)	0.0635	0.0508	0.0508	0.0635	0.0508	–
Spring rate (N/m × 10^3^)	15.1	27.5	50.1	103.1	235.7	–
Max load (*N*)	413.7	578.3	778.4	1641.4	2246.4	–

**Figure 4:**
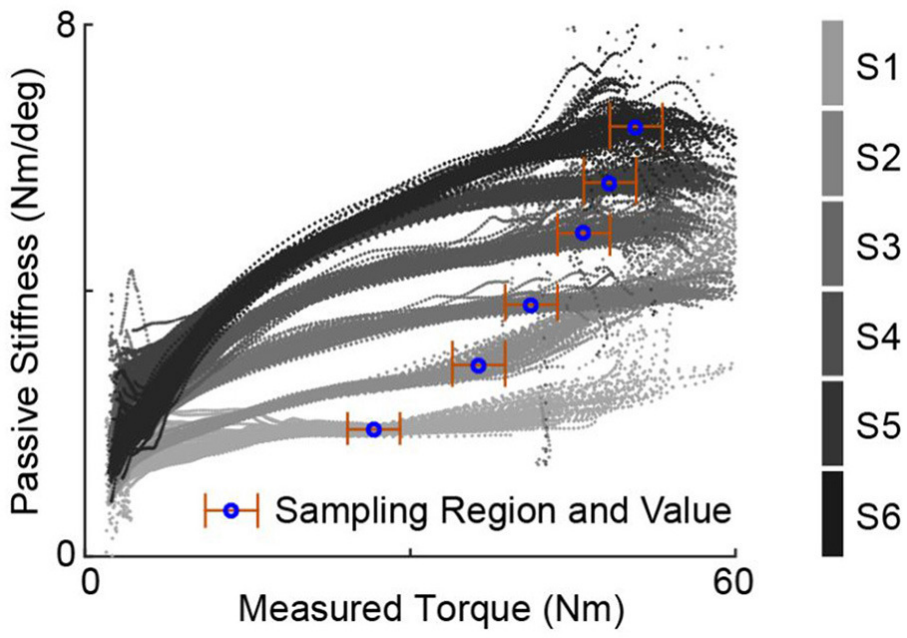
Example instantaneous passive stiffness values of passive walking sessions plotted against the measured torques for various spring configurations, one session for each. One experiment session consists of one-hundred steps with motor position fixed. The stablized passive stiffness value for one session is defined as the median of the values over a stabilized region. Note that the stablized region for each stiffness configuration was chosen based the average of multiple sessions, and the effective passive stiffness of the one stiffness configuration is defined as the mean of stabilized stiffness values across all these sessions. This figure only shows the data from one example session for each configuration, therefore, the “stablized region” may seems to have big variation.

The effective passive stiffness values of various spring configurations, *K*_*t*_, are evaluated based on passive walking experiment data. For each of six passive stiffness configurations, the human subject walks on the treadmill for at least one hundred steady steps wearing the exoskeleton with the motor position fixed at the position where force starts to be generated with the subject standing in neutral position. Such walking sessions were repeated multiple times for the same passive stiffness along the study. For each session of one hundred steps, the instantaneous value of passive stiffness at each time stamp was calculated and presented in relation to the measured torque values. Figure 4 presents such plots of passive walking sessions for different spring configurations, one session for each configuration. Median of the instantaneous passive stiffness values within the stabilized region was defined as the stabilized passive stiffness value of the session. For any spring configuration, its stabilized region is defined as a 5.65 Nm torque range, within which the change of trend for the instantaneous passive stiffness averaged over all sessions is minimum. The mean of the stabilized passive stiffness values across multiple experimental sessions for the same passive stiffness configuration was then used as its effective passive stiffness value.

#### 2.6.3. Definition of difference in desired and passive stiffness values

The difference between the desired and passive stiffnesses is an important index since Hypotheses 1 and 2 state that the optimal passive stiffness for torque tracking equals the the desired quasi-stiffness and torque errors are closely related to the difference between the two. In analyzing our experimental results, this value is defined as the algebraic difference between the desired and passive values, i.e., *K*_*t*_ − *K*_*des*_.

#### 2.6.4. Experiment procedures

The key to be able to compare the influence of passive stiffness on torque tracking performance under a fixed desired quasi-stiffness is to evaluate the “best” tracking performance under each passive stiffness configuration. We did so by experimentally evaluate the tracking errors of multiple experimental sessions, each with different feedback control gains. The lowest error across these trials was then assigned as the estimate of the actual optimal performance with this passive stiffness.

For each combination of desired and passive stiffnesses, the initial session had fairly low proportional and damping gains. The gains were gradually increased across trials until perceptible oscillations were detected with maximum damping gain. Depending on the initial gains and step sizes of gain tuning, number of trials varies for each stiffness combination. Sometimes, the gains are lowered in the final sessions to achieve better gain tuning resolution. On average, around ten trials were conducted for each stiffness combination.

Identification of the best torque tracking performance for a specific desired and passive stiffness combination is crucial. The step-wise root-mean-squared (RMS) torque tracking errors averaged over the one hundred steady steps for each experimental trial was calculated as its performance indicator. For each combination of desired and passive stiffnesses, the RMS error values of all trials with different gains were compared. The lowest of them was recorded as the estimate of optimal torque error for the corresponding stiffness combination. The control gains of the corresponding data set were recorded as the estimates of optimal control gains.

**Figure 5:**
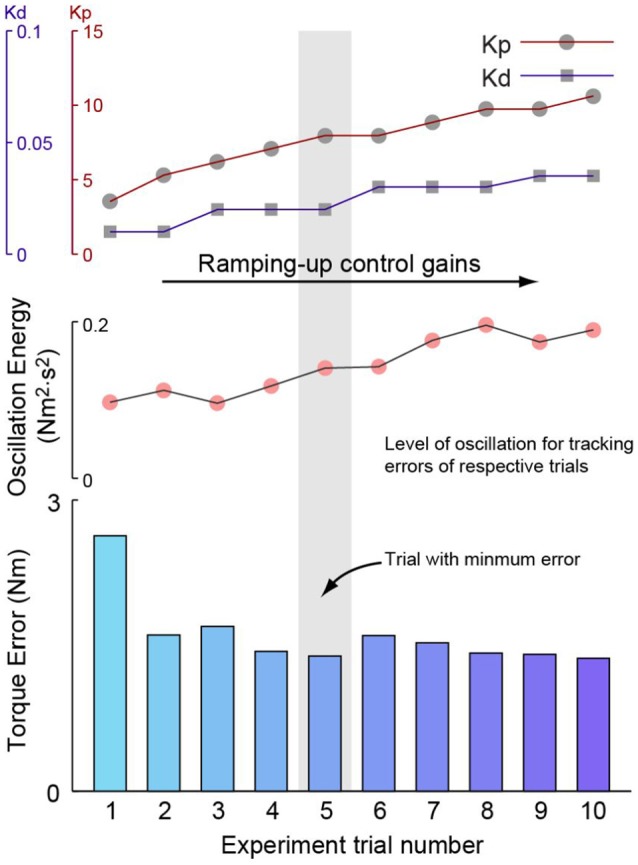
Demonstration of the experimental procedure for one example combination of desired and passive stiffness. For stiffness combination, the first experiment session starts with a moderate set of proportional and damping injection gains. For the subsequent sessions, each of which includes at least one-hundred steady strides, the proportional gain was firstly increased until oscillations became noticeable. Then, damping injection gain was increased until oscillations were reduced to be imperceptible. Proportional and damping injection were then increased alternatively until perceptible oscillations seen with maximum damping injection gain. The root-mean-squared torque tracking errors of each session was then calculated and compared against one another. The best-performed session was identified with the lowest error and its tracking error and control gains are then registered as the estimates of optimal tracking performance and optimal control gains for this specific stiffness combination. The level of oscillation for each session is also displayed. It is seen that with the increase of control gains, torque tracking errors first drop, but later increase due to increasing oscillations.

Then, the lowest torque tracking errors and the control gains of corresponding experimental sessions for all stiffness combinations were investigated against the difference between desired and passive stiffness values to test the hypotheses. This process is demonstrated in Figure 5, which presents the control gains, experimental sequence, resulting RMS torque errors and the corresponding oscillation levels of measured torques for each data set with one combination of desired and passive stiffness.

#### 2.6.5. Level of oscillation

**Figure 6:**
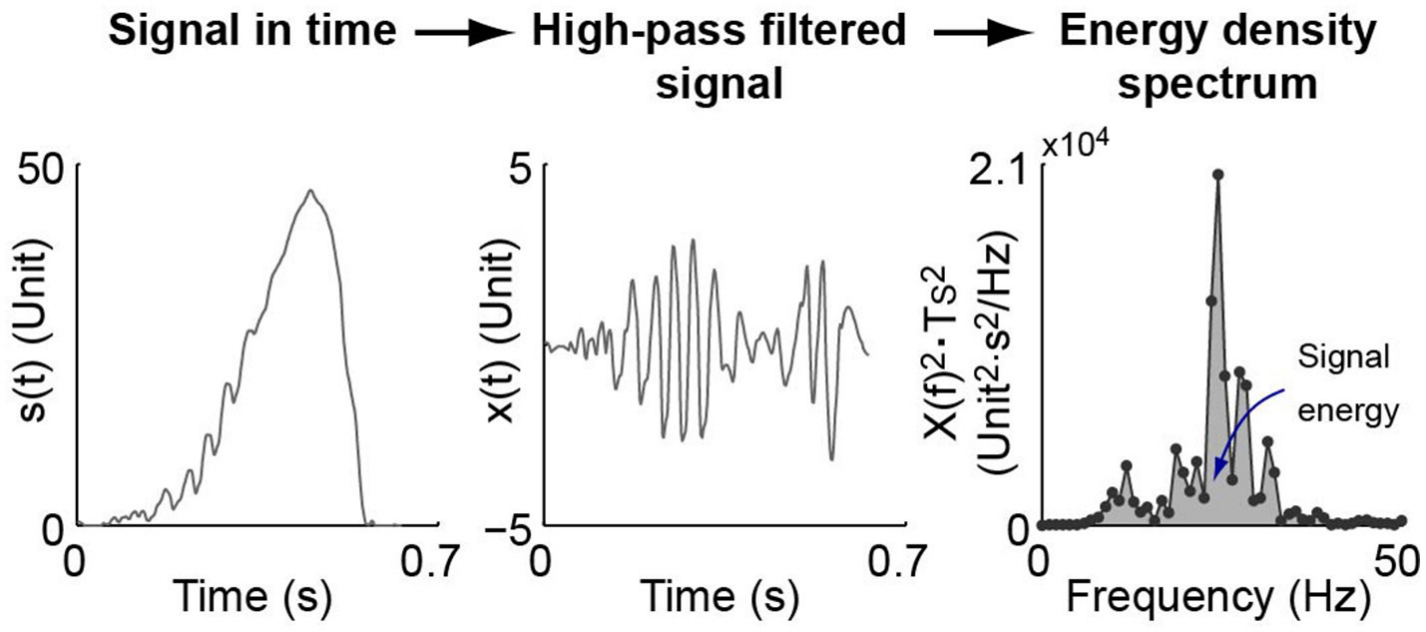
Demonstration of signal level of oscillation definition. A signal within one stance period in time domain, *s*(*t*), is firstly high-pass filtered at 10 Hz. The filtered signal, *x*(*t*), is converted to frequency domain using Fast Fourier Transform. The resulting signal in frequency domain, *X*(*f*), is used to construct the energy spectral density as $X{\left(f\right)}^{2}\cdot T_{s}^{2}$, in which *T*_*s*_ is the sampling period of the signal. The total energy of oscillation of signal *s*(*t*) is then the integral of the energy spectral density. The level of oscillation of a signal in one experiment session is then achieved by averaging that of every stride.

The level of oscillation included in Figure 5 is an indicator defined to show the amount of oscillations in the control results of each experiment session. As exemplified in Figure 6, oscillation level of one experiment session is defined as the mean stride-wise oscillation energy of the torque tracking error signal above 10 Hz. The total oscillation energy of a signal *s*(*t*) within one stance period is achieved by firstly high-pass filtering it at 10 Hz. The filtered signal, *x*(*t*), is converted to frequency domain using Fast Fourier Transform. The resulting signal in frequency domain, *X*(*f*), is used to construct the energy spectral density as $X{\left(f\right)}^{2}\cdot T_{s}^{2}$. The total energy of oscillation of signal *s*(*t*) is then calculated as the integral of the energy spectral density. The level of oscillation of a signal in one experiment session is then achieved by averaging the stride-wise torque error oscillation energy.

## 3. Results

The resulting stabilized passive stiffness values are listed in Table 4. Although the reported spring stiffness values span a huge range (Table 3), the actual maximum value is only around three times the minimum due to the existence of the Bowden cable synthetic rope in series with the spring, which exhibits the property of a non-linear spring.

**Table 4:** List of measured stabilized passive stiffness values.

**Passive stiffness ID**	**S1**	**S2**	**S3**	**S4**	**S5**	**S6**
*K*_*t*_ (Nm/deg)	1.9	2.8	3.7	4.7	5.6	5.9

Over five hundred successful experiment trials, each identified by a unique combination of control gains, desired curve and passive stiffness, were conducted and used for data analysis.

**Figure 7:**
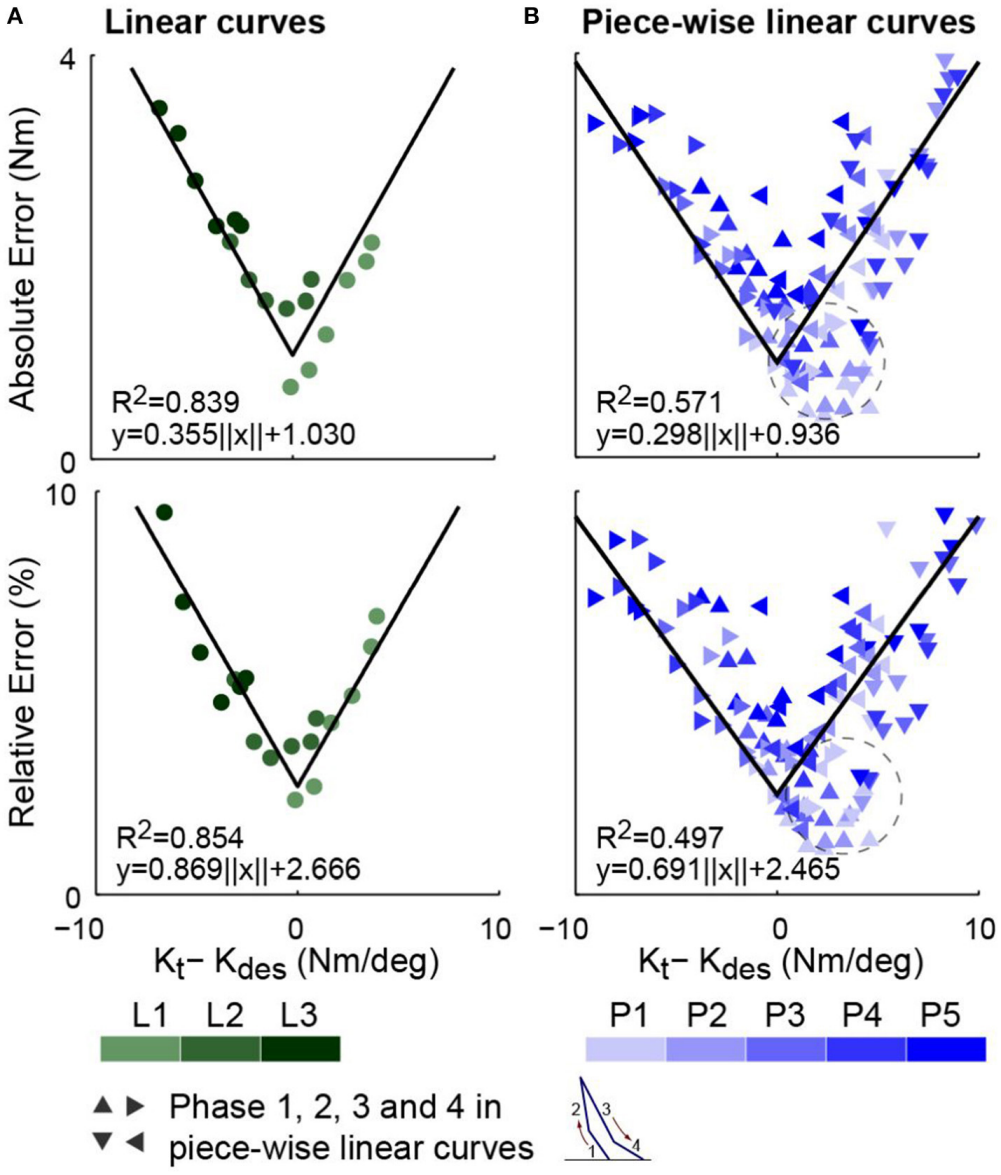
Estimates of optimal torque tracking errors, i.e., those of the trials with minimum errors, for all combinations of passive stiffnesses and desired torque curves. **(A)** Torque errors for linear curves. **(B)** Torque errors for piece-wise linear curves with four phases presented separately. Row one presents the absolute errors. Row two presents the percentage of absolute errors relative to the peak desired torque of the corresponding experiment session. For both curve types, experimental estimates of minimum torque tracking errors show fairly strong linear correlation with the absolute values of the difference between the passive stiffness and the desired stiffness, ||*K*_*t*_ − *K*_*des*_||, which agrees with Hypothesis 1 and Hypothesis 2.

Estimated optimal tracking errors, i.e., the RMS torque errors of the data sets with minimum errors, for linear curves are approximately linearly related to the absolute difference between desired and passive stiffness values as hypothesized by Hypothesis 1 and 2 (Figure 7A). It can be observed that torque errors show strong linear correlation with the absolute value of *K*_*t*_ − *K*_*des*_ in cases of both individual desired curves and all curves combined. Minimum torque errors for all curves combined are linearly related to a translated absolute value of *K*_*t*_ − *K*_*des*_, i.e,

(40) $$\begin{array}{l} e_{\tau ,opt,RMS}=a\cdot ||K_{t}-K_{des}||+b \end{array}$$

with a coefficient of determinant *R*^2^ = 0.839 at a slope of *a* = 0.355 for the absolute ones and *R*^2^ = 0.854 at *a* = 0.869 for the relative ones.

For piece-wise linear curves, the RMS torque errors of separate phases for data sets with minimum errors are also well correlated to their corresponding differences between the passive and desired stiffnesses (Figure 7B). The absolute and relative errors for all phases and curves combined are fitted with the translated absolute value of *K*_*t*_ − *K*_*des*_ with coefficients of determination *R*^2^ = 0.571 and *R*^2^ = 0.497 respectively. The slopes are *a* = 0.298 and *a* = 0.691. Note that for phases 1, 2, and 4, a fixed desired slopes exists in all steps of all data sets for the same desired curve. However, for phase 3, since the peak dorsiflexion angle is different for each step of each data set, the desired slope for a trial with minimum errors is defined as the phase 3 slope in its average stride.

For the cases of both curve types, results (Figure 7) agree with Corollary 2, and thus both Hypothesis 1 and 2, which serve as bases for it.

**Figure 8:**
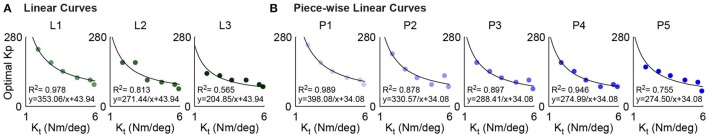
Values for the estimates of optimal proportional gain, *K*_*p*_, i.e., those of the trials with minimum errors, of various passive stiffness configurations show fairly strong inverse proportional correlation with the respective passive stiffness values for all desired curves (*R*^2^ ≥ 0.565), which agrees with Hypothesis 3. **(A)** Relationship between optimal proportional gain and passive stiffness for linear curves. **(B)** Relationship between optimal proportional gain and passive stiffness for piece-wise linear curves.

Control gains show interactions with desired and passive stiffnesses (Figure 8). The proportional gains of the trials with minimum errors for all desired curves, which are the experimental estimates of optimal proportional gains, saw strong inversely proportional correlation with passive stiffness values (*R*^2^ ≥ 0.565). For each desired curve, data were fitted into a curve with the same format as Equation (30), in which the same λ values were asserted for all curves of the same type, i.e., linear or piece-wise linear. This result agrees with Hypothesis 3, which is based on Conjecture 1.

**Figure 9:**
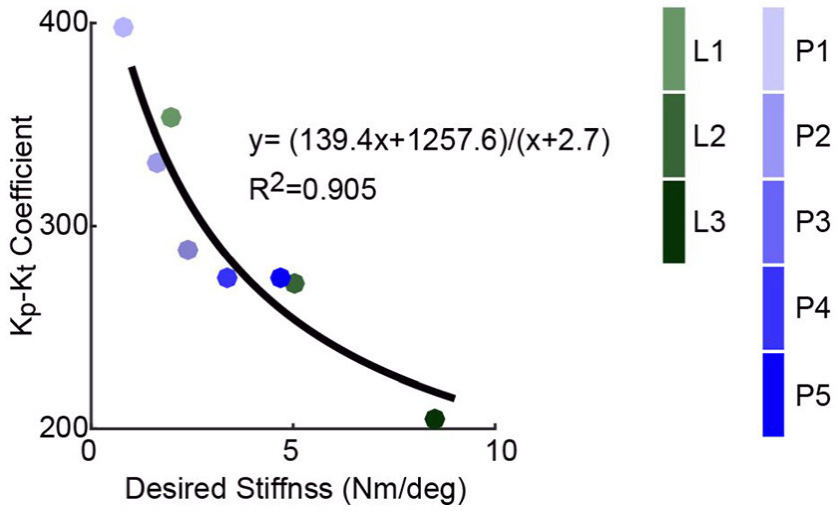
Values of gain-stiffness inverse correlation coefficient, σ, which were achieved by curve fitting in Figure 8, are inversely proportional to the desired stiffness values of various curves, which agrees with Hypothesis 4. The effective desired stiffness of a piece-wise linear curve is defined the mean of desired stiffness values across four phases averaged over all the six best-performed data sets, one for each passive stiffness configuration.

The *K*_*p*_ − *K*_*t*_ coefficient, σ, as identified in Figure 8, was also seen to be inversely proportional to the desired stiffness (Figure 9), which agrees with Hypothesis 4 based on Conjecture 1. Note that for each piece-wise linear curve, its effective desired stiffness is defined the mean of phase-wise desired stiffness values averaged over all the six best-performed data sets, one for each spring configuration.

## 4. Discussion

This paper investigates the existence of an optimal passive stiffness that benefits torque tracking in lower-limb exoskeletons driven by series elastic actuator during walking. Based on theoretical analysis with simple transmission model, we hypothesized that to achieve best torque tracking during walking in exoskeletons, the actuator should be designed such that its passive stiffness, defined as the slope of device torque-angle relationship with motor position fixed, matches the desired quasi-stiffness. We also hypothesized a fixed maximum commendable torque error changing rate that leads to the inverse proportional correlations between optimal proportional gains and passive stiffness values for a fixed desired curve. These hypotheses were highly agreed by a large amount of walking experiment data.

Although only a simplified model of the transmission sub-system was considered, torque tracking results in Figure 7 for linear curves highly agrees with Hypothesis 1 and 2. However, the phase-wise errors for piece-wise linear curves show slightly less agreement with the hypothesis. One reason is that the control gains were optimized based on full-step instead of phase-wise performance. According to the interactions between optimal proportional gains, desired stiffness and passive stiffness presented in Figure 8, for the same passive stiffness configuration, a larger desired stiffness results in a smaller optimal proportional gain. However, the level of oscillations and step-wise root-mean-squared torque errors are collectively determined by tracking performance of all four phases. Therefore, the optimal proportional gain for a piece-wise linear curve is expected to be higher than the optimal gain for the phase with largest desired stiffness and lower than the one with smallest. This means that the phase-wise torque errors in piece-wise linear curves are noisier than those of linear curves. Another issue was that for some phases, for example phase 1 of P1, P2, and P3, the desired torques were very low. Since the Bowden cable rope was still slacking at the beginning of stance, the effective passive stiffness values were actually a lot smaller than the stabilized values we used in data analysis. Therefore, many data points as circled in Figure 7B should be shifted to the left, which will improve the fitting. We also attempted to evaluate the effective difference in desired and passive stiffness, *K*_*t*_ − *K*_*des*_, of piece-wise linear curves for full steps and present torque errors in a way similar to the linear curves in Figure 7A. One way we tried is to generate the effective desired stiffness of piece-wise linear curves by linearly fitting the average stride and use it to then calculate *K*_*t*_ − *K*_*des*_. Another method tried is to calculate the difference as the area between desired stiffness vs. torque curve and passive stiffness vs. torque curve. For both cases, the relationships between torque errors and effective stiffness differences showed significantly less agreement with Equation (28) than Figure 7B. This suggest that when Hypothesis 1 and 2 are used in guidance to choose passive stiffness, the concerning desired stiffness value *K*_*t*_ should be the instantaneous values instead of a collective determined values.

Meanwhile, there are other factors that add noise and complexions to the data, which causes imperfection in curve fitting and non-zero torque errors at *K*_*t*_ = *K*_*des*_ as shown in Figure 7. First factor is the experimental method used. The optimal performance of each desired and passive stiffness combination were achieved by gradually increasing proportional and damping injection gains until perceptible oscillations happen with maximum damping gains. There are multiple noise sources cased by this test scheme. The most obvious one is the testing of discrete gain values, which results in the fact that the gain values of the best-performed experiment session are mostly not the optimal gains but actually values close to them. Second, increase of control gains stops when the oscillations become noticeable for the subject, which makes the stopping criteria subjective. Although the same subject was use throughout all experiments, adaptation and subject physical condition both affect the subject's judgment of when discomfort starts, which potentially leads to higher gains tested when the subject has higher tolerance. In some cases, increase of gains stop before the torque errors hit minimum due to inability of human to tolerate oscillations, which affects the estimation of minimum torque errors and optimal control gains. Besides subjectivity of testing, actual changes in system dynamics also causes noise in data. These changes include subject physical condition across experiment sessions, human body instant mechanical properties changes due to muscle tensioning, gait variations and movements in human-exoskeleton interface. Another reason that leads to imperfection in the alignment between theory and experiment results is the employment of a highly simplified system partial model. Due to the presence of non-linear, uncertain, highly complex and changing dynamics, a lot of system features were not captured in the theoretical hypothesis. One complication that contributed was the non-linear property of the system passive stiffness due to the slow stretching property of the Vectran® cable as demonstrated by Figure 4. Due to the unstructured changes of passive stiffness between different loads and trials, only one stabilized value was used for each passive stiffness configuration. Another feature that causes complication into system dynamics but was not accounted for in theoretical analysis was the highly non-linear, complex and changing frictions in Bowden cable. Besides, we made the assumption of immediate perfect motor position tracking, which is not true in practical cases due to the limitation of motor velocity. This greatly contributed to the fact that when the passive stiffness matches desired stiffness, i.e., *K*_*t*_ = *K*_*des*_, torque errors are above zero under optimal control conditions.

Regardless of the various approximations made in various hypotheses, the results presented in Figures 7–9 support them with fairly strong correlations. The conjecture of a fixed bandwidth and thus a maximum torque error tracking rate, ė_τ, max_, as a limit for proportional gain increase suggests a potential way of systematic gain tuning when desired or passive stiffness is changed for the same subject. Due to the dependence of this maximum error changing rate on full system dynamics, it is expected to be subject-dependent for the same motor system. However, since the primary goal of this paper is the identification of an optimal passive stiffness for a desired stiffness, only one subject is used. Therefore, how the maximum tolerable torque changing rate vary among subjects remains a direction of future work.

The results of this study can be used as guidances to more efficient hardware and controller configuration for better torque tracking perforemance in lower-limb exoskeletons. For example, with a fixed desired torque-angle relationship required by one particular application, the passive stiffness of the series elastic actuator can be changed by switching the passive compliant element to match the effective desired stiffness. Then, with optimal control gains for one desired and passive stiffness combination determined through experiments on one subject, when desired stiffness changes due to application requirments, or passive stiffness changes due to hardware limitation, the optimal control gains can be estimated based on their relationships with the two stiffness values. The applications of this study will faciliate more effective and efficient torque control in lower-iimb exoskeletons and improves the performance of these devices in experimental, rehabilitation and also assistance senarios.

## 5. Conclusions

This paper hypothesizes and confirms by experiments that the optimal passive stiffness for lower-limb exoskeleton torque tracking corresponding to the desired quasi-stiffness, which is the slope of desired torque-angle relationship. The minimum torque tracking errors are shown to be linearly related to the difference between the desired and passive stiffnesses. This paper also hypothesizes a potential maximum torque error tracking rate and therefore a maximum control bandwidth that prevents the proportional feedback gain from increasing infinitely. This was also supported by experimental data. These results provide guidance for passive stiffness selection of lower-limb exoskeletons and other walking related robots for a fixed desired stiffness. They also provide guidance for optimal gain tuning in case of changing passive or desired stiffness.

## Ethics statement

This study was carried out in accordance with the recommendations of guidelines of the Office of Human Research Protection (OHRP) and other federal regulatory agencies with written informed consent from all subjects. All subjects gave written informed consent in accordance with the Declaration of Helsinki. The protocol was approved by the Carnegie Mellon University IRB.

## Author contributions

JZ and SC contributed equally in theory development and experiments for this study.

### Conflict of interest statement

The authors declare that the research was conducted in the absence of any commercial or financial relationships that could be construed as a potential conflict of interest.
